# Cellulose-Based Scaffolds: A Comparative Study for Potential Application in Articular Cartilage

**DOI:** 10.3390/polym15030781

**Published:** 2023-02-03

**Authors:** Rachel Cordeiro, Rui D. Alvites, Ana C. Sousa, Bruna Lopes, Patrícia Sousa, Ana C. Maurício, Nuno Alves, Carla Moura

**Affiliations:** 1 Centre for Rapid and Sustainable Product Development, Polytechnic of Leiria, 2430-028 Marinha Grande, Portugal; 2 Veterinary Clinics Department, Abel Salazar Biomedical Sciences Institute (ICBAS), University of Porto (UP), Rua de Jorge Viterbo Ferreira, No. 228, 4050-313 Porto, Portugal; 3 Animal Science Studies Centre (CECA), Agroenvironment, Technologies and Sciences Institute (ICETA), University of Porto, Rua D. Manuel II, Apartado 55142, 4051-401 Porto, Portugal; 4 Associate Laboratory for Animal and Veterinary Science (AL4AnimalS), 1300-477 Lisbon, Portugal; 5 Associate Laboratory for Advanced Production and Intelligent Systems (ARISE), 4050-313 Porto, Portugal; 6 Applied Research Institute (i2A), Polytechnic Institute of Coimbra, Rua da Misericórdia, Lagar dos Cortiços–S. Martinho do Bispo, 3045-093 Coimbra, Portugal

**Keywords:** cartilage repair, PCL, cellulose, scaffold, tissue engineering

## Abstract

Osteoarthritis is a highly prevalent disease worldwide that leads to cartilage loss. Tissue engineering, involving scaffolds, cells, and stimuli, has shown to be a promising strategy for its repair. Thus, this study aims to manufacture and characterise different scaffolds with poly(ε-caprolactone) (PCL) with commercial cellulose (microcrystalline (McC) and methyl cellulose (MC) or cellulose from agro-industrial residues (corncob (CcC)) and at different percentages, 1%, 2%, and 3%. PCL scaffolds were used as a control. Morphologically, the produced scaffolds presented porosities within the desired for cell incorporation (57% to 65%). When submitted to mechanical tests, the incorporation of cellulose affects the compression resistance of the majority of scaffolds. Regarding tensile strength, McC2% showed the highest values. It was proven that all manufactured scaffolds suffered degradation after 7 days of testing because of enzymatic reactions. This degradation may be due to the dissolution of PCL in the organic solvent. Biological tests revealed that PCL, CcC1%, and McC3% are the best materials to combine with human dental pulp stem/stromal cells. Overall, results suggest that cellulose incorporation in PCL scaffolds promotes cellular adhesion/proliferation. Methyl cellulose scaffolds demonstrated some advantageous compressive properties (closer to native cartilaginous tissue) to proceed to further studies for application in cartilage repair.

## 1. Introduction

Osteoarthritis (OA) is a very complex process characterised by inflammation, synovial distension, articular cartilage (AC) loss, and bone hyperplasia [1]. A 2019 study indicates that OA is the 17th most prevalent disease worldwide, considering 369 possible diseases [2]. The most recent statistics indicate that about 250 million people are affected by this disease [3]. Furthermore, the number of people older than 60 with OA is expected to be about 2 billion in 2050 [4]. Thus, the field of tissue engineering (TE) has generated interest in the development of a long-term and effective treatment for AC repair [5].

TE uses mainly scaffolds, cells, and biochemical and/or biomechanical stimuli [6]. Scaffolds are temporary implants fundamental in the development of new tissue since they support the growth of tissues and cells [7]. To provide this support, the material that composes it is a factor to take into account [8]. Cells are also an important factor since they are fundamental for tissue maintenance. On the other hand, biochemical stimulation, i.e., growth factors, are used to support cell differentiation. Biomechanical stimulation in TE is used to improve the properties of the new tissue to be formed since this stimulation leads to increased extracellular matrix production [9].

Poly(ɛ-caprolactone) (PCL) is a semicrystalline polyester with melting temperatures around 55–60 °C, which makes it the perfect polymer for 3D extrusion [10,11]. It is the most used polymer in the TE field [12]. It is approved by the Food and Drug Administration [13], exhibits good mechanical properties, is biodegradable, has a slow degradation rate, and improves the cell differentiation and proliferation of chondrocytes [10,11,14]. However, their hydrophobicity makes cell attachment difficult [15,16,17]. Some studies blend this with natural materials to improve the biological activity in PCL scaffolds [18].

Cellulose is a natural polymer, the biopolymer most abundant in nature. The principal sources of cellulose were plants, bacteria, algae, and marine animals. In addition, it is renewable, biocompatible, and biodegradable. The use of the term biodegradable for cellulose, when applied to the human body, is from a perspective of comparison with other polymers since the human body does not have cellulases to degrade it [19]. The structure of cellulose is a repetition of glucose molecules joined by a β(1→4) bond with a high possibility of obtaining derivates since the OH groups can be replaced by other chemical groups [20,21].

The use of cellulose in scaffold manufacturing for application in different tissues (skin, bone, vessels, ocular tissue, and cartilage) has been growing [22]. This growth is due to the several advantages cellulose offers, such as the diversity of sources, different properties depending on the source, good cell adhesion, its crystalline and amorphous structure, and the consequence of the several arrangements in the chains, and its mechanical properties [23]. There are various polymorphs of cellulose, such as cellulose I, II, III, and IV. However, only cellulose II is completely biodegradable and appropriate for tissue repair, as it has no crystalline phase [24]. The interest in this polymer for cartilage TE is based on its chemical similarity to AC collagen fibres [25].

There are studies in the literature referring to the combination of PCL with cellulose with different scaffold manufacturing techniques [4,14,26]. Alemán-Domínguez (2018) developed scaffolds for bone TE with PCL and microcrystalline cellulose (McC) [4]. Four cellulose concentrations were evaluated, 0, 2, 5, and 10% (*w*/*w*), to verify the influence of its addition to PCL. The results showed that the scaffolds produced with 2% McC demonstrated the most suitable properties for the bone application. In the previous study, the same authors studied the influence of cellulose derivative, carboxymethyl cellulose, on PCL scaffolds [14]. Three concentrations were tested, 0, 2, and 5% (*w*/*w*), and, once again, 2% of carboxymethyl cellulose on PCL scaffolds proved to be the concentration with the highest potential for the same application. This cellulose was previously studied as its biomineralisation capacity had been reported [27]. Methyl cellulose (MC) is also a cellulose derivative. Its non-toxic, biocompatible, and gelling properties lead us to believe that it is a biomaterial with great potential to be included in scaffolds for cartilage repair. Roushangar (2018) included MC in scaffolds for cartilage repair and demonstrated that these had a high efficiency of living chondrocytes. In particular, MC increased the viscosity leading to the desired pore size [28]. Moreover, MC is considered a bioink since it can be used as a gel [29].

Kotcharat (2021) also studied the influence of bacterial cellulose in PCL implants for wound dressing [26]. Results proved that bacterial cellulose improves the swelling properties of the scaffold.

A study by our group comparing scaffolds with McC and corncob cellulose (CcC) indicated that CcC could be a sustainable alternative to wood cellulose. Furthermore, it was proven that cellulose promotes cell proliferation [30]. However, it has not been established which concentration is ideal for replacing commercial wood cellulose. In this study, PCL and McC, CcC and MC scaffolds were manufactured by fused deposition modelling technique. The scaffolds were manufactured with three different concentrations of each cellulose (1%, 2%, and 3%). They were characterised and compared with pure PCL in terms of morphology, chemical and mechanical properties, enzymatic degradation, and cell viability.

## 2. Materials and Methods

### 2.1. Materials

Poly(ε-caprolactone) (PCL) (Perstorp, Warrington, UK), with 6500 g/mol molecular weight, was used as a base-polymer. The different commercial celluloses used were microcrystalline cellulose (Avicel PH102, FMC Biopolymer, Braine l’Alleud, Belgium) and methyl cellulose (viscosity 15cP, Acros Organic, Tokyo, Japan). The corncob cellulose was kindly provided by the College of Agriculture of the Polytechnic Institute of Coimbra (ESAC-IPC) (Coimbra, Portugal). For the preparation of the polymeric mixture and for the enzymatic degradation analysis of scaffolds, dimethylformamide (DMF) (Chem-Lab, Zedelgem, Belgium), acetone (HoneyWell, Offenbach, Germany), lysozyme (Sigma-Aldrich, Oakville, ON, Canada), and phosphate-buffered saline (PBS) (Sigma Aldrich, Saint Louis, MO, USA) were used.

### 2.2. Preparation of Polymeric Mixtures

To manufacture the scaffolds, it is necessary to develop a composite, binding the PCL and cellulose. This mixture was prepared by solvent casting technique. Firstly, 17% (*w*/*w*) of cellulose solution in acetone:DMF (2:1) (*w*/*w*) was prepared at 50 °C under constant stirring (150 rpm) for 30 min. Then, PCL and DMF (1:3) (*w*/*v*) were added to the cellulose mixture. The final solution was placed under constant stirring (150 rpm) at 50 °C for 48 h. The mixtures were produced in 3 different concentrations, 1%, 2%, and 3% of cellulose:PCL (*w*/*w*). PCL: (acetone:DMF) (1:3 (*w*/*v*)) was used as a control sample. 

Solutions were deposited in Petri dishes to dry at room temperature (RT) for 7 days to obtain the mixtures in the solid phase (membranes). In total, 10 mixtures were made, classified depending on the type and the proportion of cellulose used (Table 1).

### 2.3. Infrared Characterisation

The ATR–FTIR spectra were recorded to confirm that all DMF had been evaporated from the dried samples. A Bruker Alpha-P FTIR spectrometer (Billerica, MA, USA) was used in the absorbance mode, with ATR platinum–diamond coupling. The samples were analysed at RT, 2 cm^−1^ spectral resolution, at 64 scans per sample, and in the range 4000–400 cm^−1^. The analysis was performed in triplicate, and the sample portion for analysis was randomly selected.

### 2.4. Scaffolds Manufacturing

Scaffolds were obtained by extrusion using the Fused Deposition Modelling (FDM) technique in Bioextruder (Portuguese Patent N. 104247, 2010), equipment developed by CDRSP, with a 0.4 mm nozzle needle. The membranes previously prepared were cut into small pieces and placed inside the extruder tank. The 3D structures were designed as cylinders, so their cross-section had a diameter of 10 mm, a fibre diameter of 300 μm, composed of 10 layers, and a 0°/90° lay-down pattern. The equipment and the extrusion parameters varied from sample to sample (Table 2) to obtain the desired scaffold features.

### 2.5. Morphology Analysis

The morphology of the scaffolds was evaluated by microscopy (Leica DM750M Microsystems, Northbrook, IL, USA) at a magnification of 5×, and by micro-computed tomography (MicroCT), using a SkyScan 1174TM (Brucker, Kontich, Belgium). The filament and pore dimensions of each scaffold were analysed using microscopy. For this, a scaffold was randomly selected and 5 images were captured at different points. The measurements were performed in 5 different pores and filaments in each image through the ImageJ v.1.53t software. For the MicroCT, the parameters used for the digitalisation of the samples were: a step of 0.6 degrees around the medio-lateral axis, an acceleration voltage of 50 kV; a beam current of 800 µA; an exposure time of 4000 ms; a pixel size of the image of 14 µm; and no filters were used. The 3D reconstruction was performed in NRecon v.1.7.3.1 software. The morphological parameters, porosity, and pore interconnectivity (*N* = 3) were calculated with CT-Analyser v.1.17.7.2 software.

### 2.6. Mechanical Tests

Resistance at compression and tensile was carried out to evaluate the effect of different celluloses and percentages in scaffolds and filaments produced by extrusion. According to ASTM standards, a compression test was performed using universal testing equipment (Instron 5544, Canton, MA, USA). The extension rate was 1 mm/min. During the test, samples were compressed to at least 50% of their height to assess the material’s behaviour upon compressive forces to guarantee that the yield strength is not achieved, i.e., applied forces are within the elastic region. All mechanical testing were realised using dried scaffolds. The compression modulus was calculated by the slope of the linear region in the stress-strain curve (elastic region).

Regarding the tensile test, it was performed using a texturometer (TA.xTplusC, Stable Micro Systems, Godalming, UK). Tensile testing was carried out using a 50 Kg (≈490 N) load cell at a speed of 10 mm/s with an initial spacing of 20 mm between strings. Filaments tested were cut with 30 mm of height and presented a slight variation in diameter (Table 3). The test was performed in triplicate, and all filaments were subjected to force until broken.

### 2.7. Enzymatic Degradation Assay

An enzymatic analysis using 1.5 mg/mL lysozyme in PBS (pH = 7.4) was performed to evaluate the degradation of scaffolds produced. The medium with lysozyme was stirred for 10 min at 150 rpm, added to each well, and incubated at 37 °C in an orbital shaker at 100 rpm. The enzymatic medium was refreshed every 3 days. On the 0th, 1st, 7th, 28th, and 42nd days, scaffolds were removed from the wells and dried for 3 days at RT. All scaffolds were weight before and after degradation. As a control, scaffolds were incubated under the same conditions without enzymes. Tests were performed in triplicate.

### 2.8. Cellular Population and In Vitro Culture

Human Dental Pulp stem/stromal cells (hDPSCs) previously characterised by J. M. Campos et al. (2019) [31], were acquired from AllCells, LLC (Cat. DP0037F, Lot N°DPSC090411-01) and cultured in Mem α, GlutaMAX™ Supplement, no nucleosides (Gibco, 32561029), supplemented with 10 mM HEPES buffer solution (Gibco, 15630122), 100 IU/mL penicillin, 0.1 mg/mL streptomycin (Gibco, 15140122), 2.05 µm/mL amphotericin B (Gibco, 15290026), and 10% (*v*/*v*) fetal bovine serum (FBS) (Gibco, A3160802). Cells were maintained under standard conditions, namely at 37 °C, with 80% humidity and 5% CO_2_, as described in Branquinho et al. (2021) [32].

### 2.9. Cytocompatibility Evaluation

The PrestoBlue^TM^ viability assay, as previously described by Alvites et al. (2021) [33], was used to determine the cytocompatibility between the hDPSCs and the different scaffolds. Before the assay, the scaffolds were sterilised using exposure to ultraviolet radiation in 20-min cycles on each surface of the biomaterials. Then, the scaffolds were placed in 24-well plates, and hDPSCs at P5 were seeded in a low-volume suspension on top of the devices at a density of 7000 cells per well/scaffold. The plates were kept under standard conditions for 1 h to maximise cell adhesion to the scaffolds, and then wells were filled with a complete culture medium. The same procedure was performed for the control group, in which cells were seeded directly onto the plate wells without contacting any biomaterial. Blank wells, i.e., without seeded cells, were considered for both groups receiving scaffolds and the control group. The determination of cytocompatibility between cells and scaffolds was determined at 4 different timepoints after incubation: 24 h, 72 h, 120 h, and 168 h. For each determination, the culture medium was removed from the wells and replaced with fresh culture medium plus 10% *v*/*v* 10× PrestoBlue^TM^ reagent (Invitrogen, A13262, Eugene, OR, USA). After 1 h incubation under standard conditions, 100 µL of the supernatant was collected from each well and transferred to the 96-well plate. Absorbances were read in triplicates at 570 and 595 nm with a Multiskan^TM^ FC Microplate Photometer (Thermo ScientificTM, 51119000, Woodlands, Singapore). After the reading, each well of the 24-well plate was washed abundantly with Dulbecco’s phosphate-buffered saline solution (DPBS, Gibco, 14190169) to remove residual PrestoBlue^TM^ reagent and received a fresh medium, returning to incubation conditions until the next timepoint.

The absorbance values obtained at 595 nm (emission wavelength) were subtracted from those obtained at 570 nm (excitation wavelength) for each well (pre-calculated absorbances). The mean values obtained in the blank wells were further subtracted from the respective pre-calculated absorbances of each experimental well. Two blank wells and four experimental wells were considered for each experimental group. The percentage of inhibition of cell viability induced by each scaffold was subsequently determined.

### 2.10. Statistical Analysis

Statistical analysis was carried out in GraphPad Prism v.8 software (GraphPad Software, Inc., San Diego, CA, USA), using one-way ANOVA, with Sidak’s test for the mechanical compression test, and Tukey’s test, for the mechanical tensile test. A two-way ANOVA with Tukey’s test was performed for the enzymatic analysis. For cell assays, results were presented as mean ± standard error of the mean (SEM). A two-way ANOVA analysis with Tukey’s test was performed. The level of statistical significance was set as 99.9% (* *p* < 0.05, ** *p* < 0.01, *** *p* < 0.001).

## 3. Results

**Figure 1:**
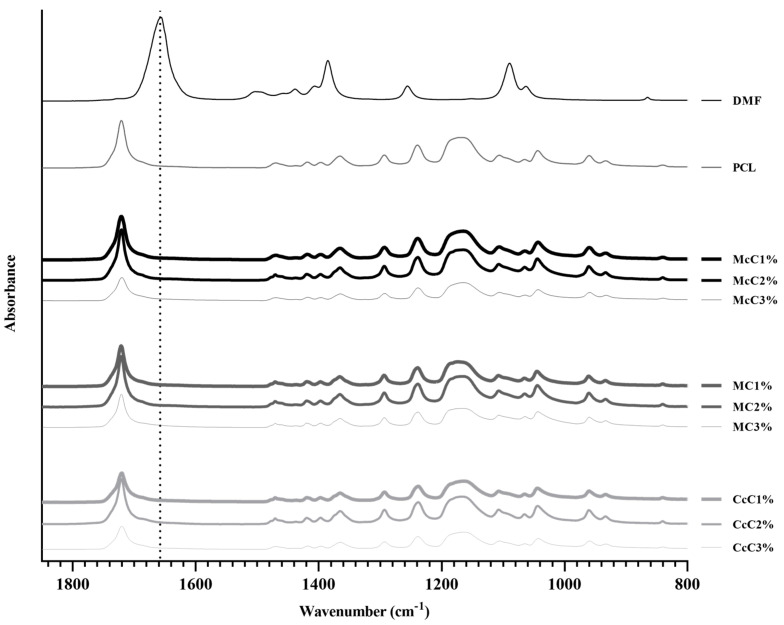
Fourier-transform infrared spectroscopy analysis of all the developed scaffolds: poly(ε-caprolactone) (PCL), microcrystalline cellulose at 1, 2, and 3% (McC1%, McC2%, and McC3%, respectively), methylcellulose at 1, 2, and 3% (MC1%, MC2%, and MC3%, respectively), and corncob cellulose at 1, 2, and 3% (CcC1%, CcC2%, and CcC3%, respectively) and pure DMF.

The PCL–cellulose composite developed in this study was combined through an organic solvent, DMF, that is expected to evaporate totality during the drying process. Therefore, an FTIR analysis was performed on all membranes to confirm that all DMF has evaporated and compared with pure DMF FTIR spectra (Figure 1), between 800 cm^−1^ and 1850 cm^−1^. The most prominent band concerns the C=O stretching of DMF (1658 cm^−1^), which does not appear in any membrane analysed, confirming the total evaporation of DMF.

**Figure 2:**
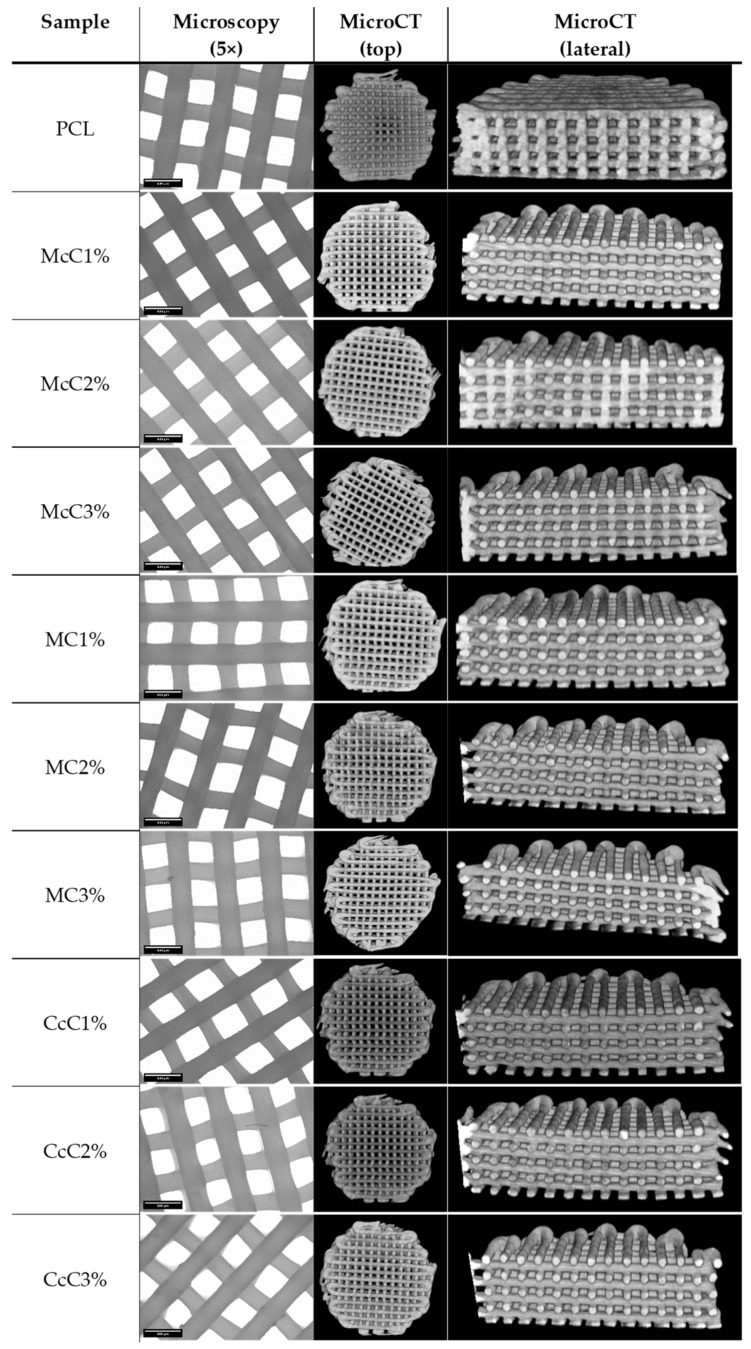
Morphological analysis of the scaffolds produced from each sample: poly(ε-caprolactone) (PCL), microcrystalline cellulose at 1, 2, and 3% (McC1%, McC2%, and McC3%, respectively), methyl cellulose at 1, 2, and 3% (MC1%, MC2%, and MC3%, respectively), and corncob cellulose at 1, 2, and 3% (CcC1%, CcC2%, and CcC3%, respectively), by microscopy, with a 5× resolution, and micro-tomography computed, observable at the top of the scaffold and in its medio-lateral region.

PCL–cellulose scaffolds were made with different cellulose and in different ratios. With this requirement, the fabrication conditions suffered some variations. However, they were optimised so that all samples had the same shape and similar dimensions. Morphological analyses were performed using microscopy and MicroCT (Figure 2) to obtain the average dimensions of each scaffold. Through optical microscopy, it is possible to obtain the filament and pore size, while through MicroCT, it is possible to obtain the porosity and interconnectivity of each scaffold.

All scaffolds were designed to have approximately a 300 µm filament and 350 µm of pore size. The CcC1% scaffold presented the thinnest filament, with 270.14 ± 9.3005 µm, whereas the McC2% scaffold presented filaments with the largest dimension (326.45 ± 11.674 µm) (Table 4). On the other hand, the smallest pore corresponds to the CcC2% scaffold and the largest to the MC2% (329.72 ± 12.525 µm and 379.57 ± 24.389 µm, respectively). The porosity of the cartilaginous matrix varies from zone to zone between 92% and 70% [34]. Porosities of the scaffolds produced are below these values, between 59.40 ± 2.616% and 65.56 ± 1.663%, with the highest value corresponding to scaffold with MC2%. The interconnectivity between pores should be as close as possible to 100% so that cells can move freely in the scaffold. Except for the McC3% scaffold, all have interconnectivity higher than 99.99%, being 100% reached by the McC1% scaffold.

**Figure 3:**
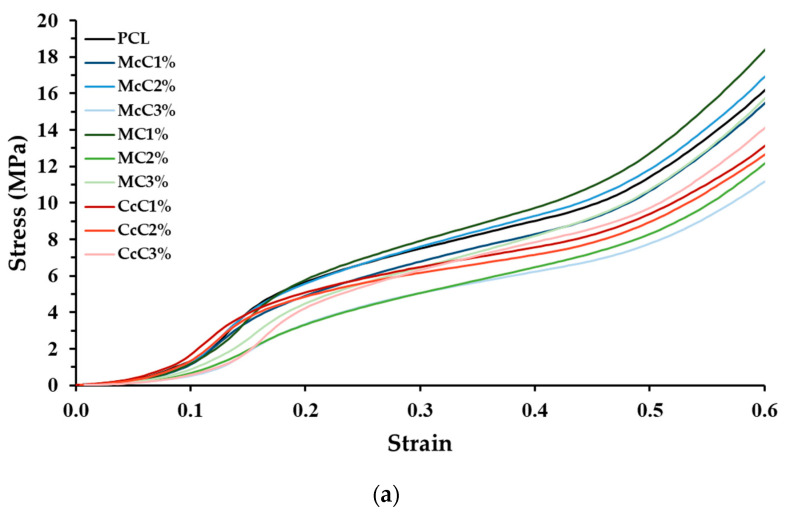

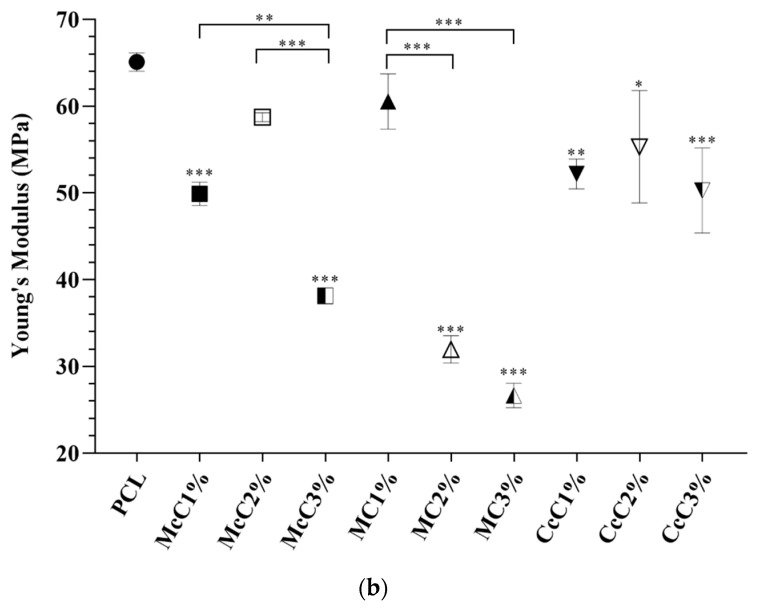
Mechanical performance of scaffolds upon compression: (**a**) Mean curves; (**b**) Compressive modulus (Mean ± SD, *n* = 3). Statistically significant differences are presented as: * *p* < 0.05; ** *p* < 0.01; *** *p* < 0.001.

Compression tests were also performed to test the mechanical strength of the scaffold produced. In the graph (Figure 3), it is visible that all scaffolds, except McC2% and MC1%, present a lower modulus than the scaffold-based (PCL). In the scaffold where commercial cellulose was incorporated, McC and MC, there is a tendency for the increase in cellulose, which leads to a decrease in strength. In the case of cellulose from residue, CcC, the resistance of the scaffold was not affected by cellulose incorporation.

**Figure 4:**
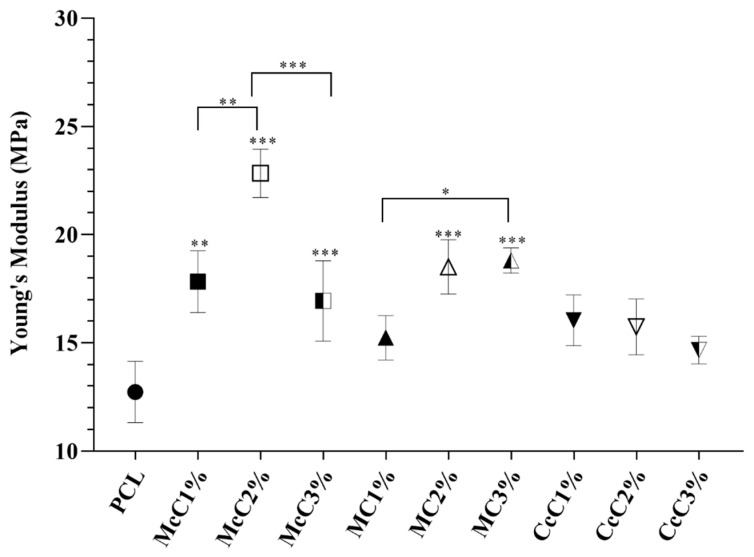
Modulus of tensile strength for filaments obtained by extrusion. (Mean ± SD, *n* = 3). Significant statistical differences are presented as: * *p* < 0.05; ** *p* < 0.01; *** *p* < 0.001.

Filament strength tests (tensile testing) were also performed. Figure 4 shows that there are also differences between commercial and waste cellulose. Fibres made from CcC show an equal resistance to the fibres only with PCL, while the fibres incorporated with McC and MC show higher resistance, except for MC1%. Further, visible changes in properties are visible within the same cellulose but in different concentrations. In McC, incorporating 2% of cellulose increases resistance, but at 3%, its resistance returns to values equal to 1%. Regarding MC, its incorporation in fibres only becomes significant at 3%, increasing its strength. As for CcC, the increase in concentration does not result in changes in its tensile strength.

**Figure 5:**
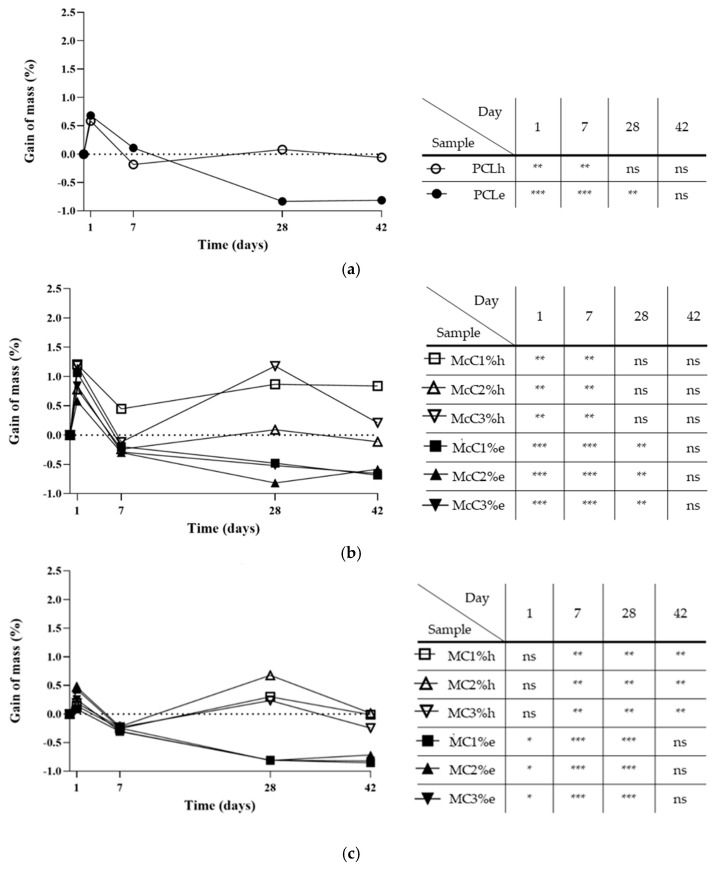

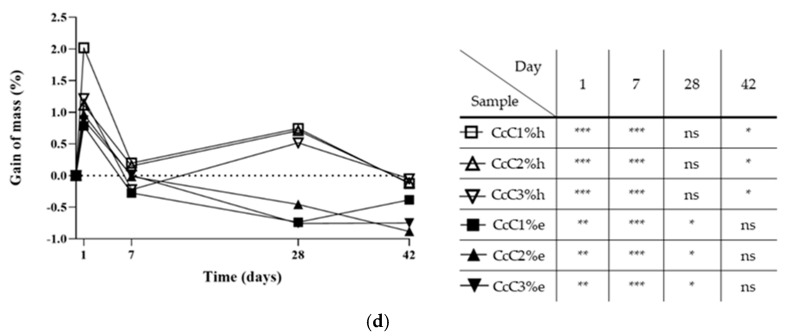
Enzymatic and hydrolytic degradation assay of the scaffolds developed for 42 days: (**a**) poly(ε-caprolactone) (PCL); (**b**) microcrystalline cellulose at 1, 2, and 3% (McC1%, McC2%, and McC3%, respectively); (**c**) methyl cellulose at 1, 2, and 3% (MC1%, MC2%, and MC3%, respectively); (**d**) and corncob cellulose at 1, 2, and 3% (CcC1%, CcC2%, and CcC3%, respectively). Hydrolytic samples are identified with an “h” and are graphically represented with an unfilled symbol (○, □, △, ▽). Enzymatic samples are identified with an “e” and graphically represented with a symbol filled (●, ■, ▲, ▼). The assay was performed in triplicates, but only mean values are presented in the graphs. Statistically significant differences are presented as: * *p* < 0.05; ** *p* < 0.01; *** *p* < 0.001. ns refers to no statistically significant differences.

An enzymatic degradation assay was also performed with lysozyme, the enzyme naturally present in cartilage. Figure 5 shows the graphs of mass gain for PCL, McC, MC and CcC scaffolds. In all scaffolds, it is possible to observe that after 1 day of testing, there was an abrupt increase in weight. By the 7th day, the weights decreased, remaining, in general, very close to a gain equal to 0. On days 28 and 42, scaffolds subjected to the enzyme presented weight loss for all types of cellulose, while scaffolds without lysozyme gained or maintained their weight. The cellulose with the highest variability in weight gain was McC, with different values among them.

**Figure 6:**
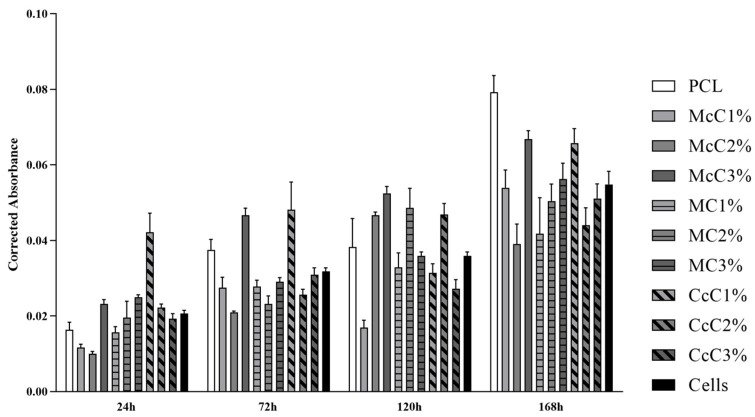
Cytocompatibility between scaffolds and hDPSCs determined at different timepoints using the PrestoBlueTM viability assay. (Mean ± Standard Error of the Mean (SEM)). Statistical differences at each timepoint can be consulted in Figure 7.

**Figure 7:**
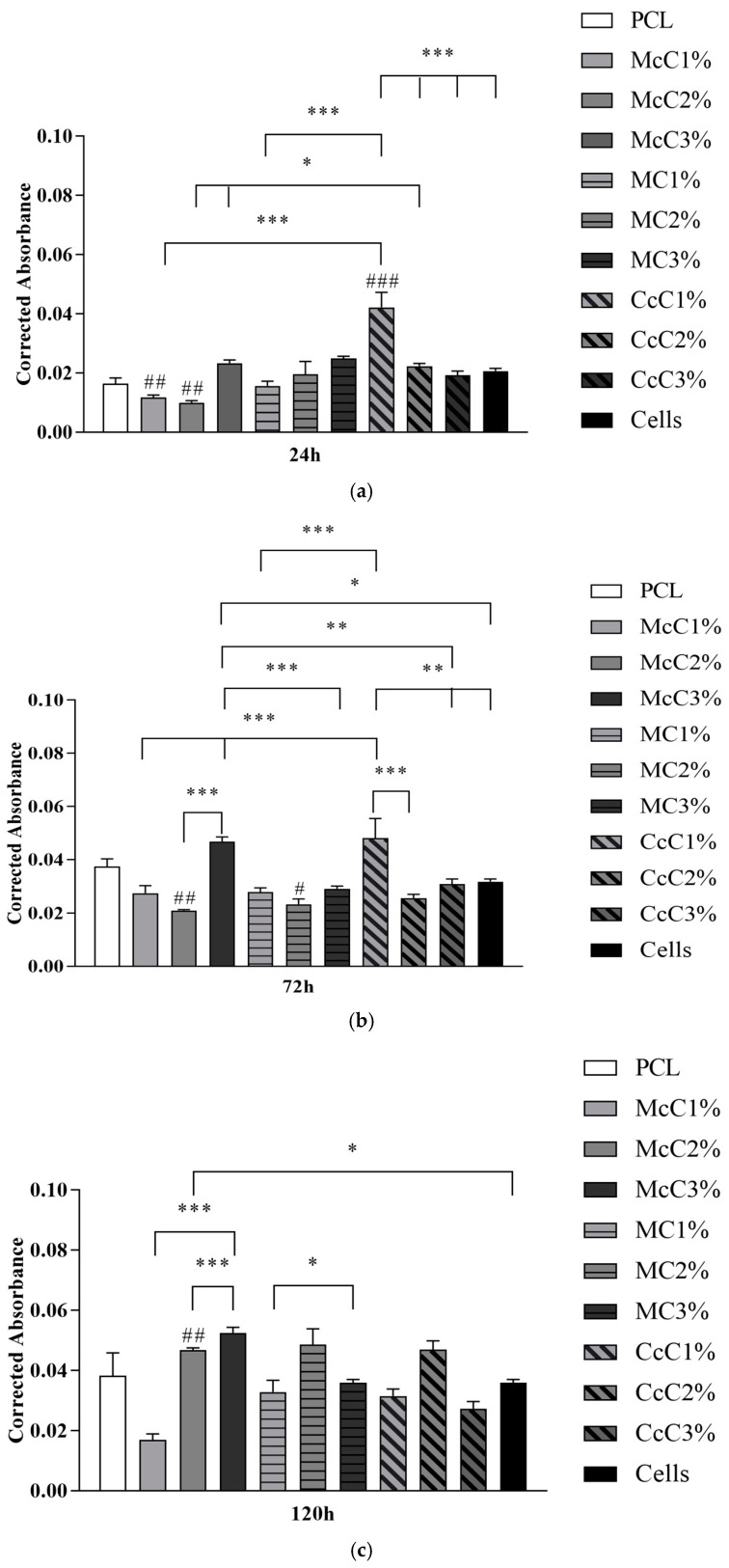

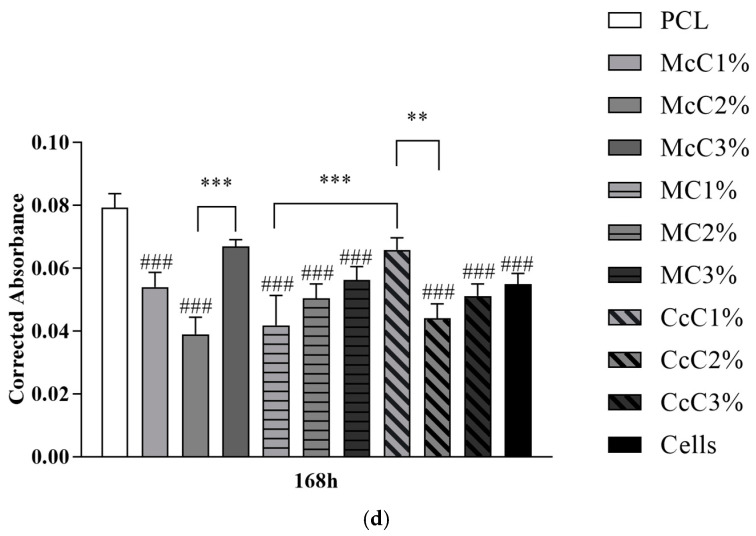
Cytocompatibility between scaffolds and hDPSCs using the PrestoBlue^TM^ viability assay, determined at: (**a**) 24 h; (**b**) 72 h; (**c**) 120 h; (**d**) 168 h. Results are expressed in Mean ± Standard Error of the Mean (SEM). Differences were considered statistically significant at *p* < 0.05. Results significances between different scaffolds were presented using the symbol (*). Differences between scaffolds and PCL were presented using the symbol (#). According to the *p*-value, *(#), **(##), and ***(###) corresponds to *p* < 0.05, *p* < 0.01, and *p* < 0.001, respectively.

Cytocompatibility analysis of the scaffolds was performed, and the corrected absorbances determined at each timepoint can be seen in Figure 6. The corrected absorbance values at each time point can be consulted in Supplementary Material. Figure 7 demonstrates the statistical differences identified between experimental groups at each time point. The results reveal a general cytocompatibility of the studied biomaterials with hDPSCs, which are more evident at more advanced timepoints.

**Figure 8:**
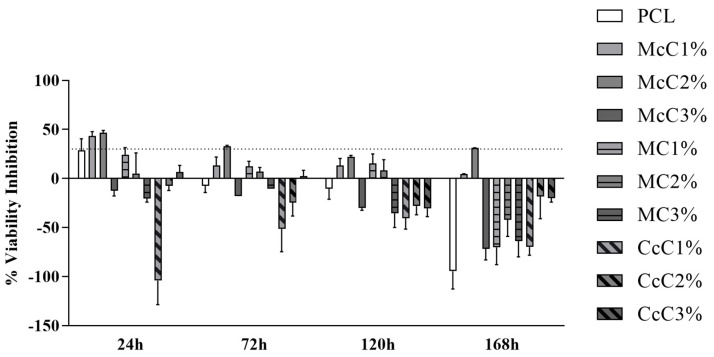
Percentage of inhibition of cell viability of each scaffold in contact with the hDPSCs. The dashed line represents the 30% inhibition limit, above which the effect is considered cytotoxic according to the ISO 10993-5:2009 guidelines.

The percentage of inhibition of cell viability is shown in Figure 8. The percentage values and the graph of each time point can be consulted in Supplementary Material. It is important to note that at some timepoints, cell viability inhibition percentage above 30% or on the borderline is observed. In these cases, biomaterials formulations are considered cytotoxic. However, this cytotoxicity is reversible in some cases, for example, at 24 h in the PCL (borderline values) and McC1% (cytotoxicity indicators). Afterwards, they indicate the absence of cytotoxicity and facilitation of cell proliferation. The McC2% scaffold can be considered the most cytotoxic.

## 4. Discussion

Previous work developed by our group [30] demonstrated that PCL–cellulose scaffolds could be a viable option to replace cartilage. In addition, CcC could be a potential substitute for McC [35]. Moreover, PCL and McC, CcC, and MC (at three different percentages in concentration) scaffolds were manufactured. MC was evaluated to assess its possibility as a viable alternative compared to the others.

In scaffold preparation and to develop the composites, it was necessary to use DMF. This organic solvent causes toxicity when in contact with cells [36] and also affects the mixing process [30]. To ensure that all DMF was removed from the membranes, an ATR-FTIR analysis was performed (Figure 1). The graph demonstrates that all DMF has been removed from membranes since the 1658 cm^−1^ band, concerning C=O stretching of DMF, does not appear in any of the membrane’s spectra. The PCL spectrum was similar to the spectra of the different PCL–cellulose composites. The concentration of cellulose included in the membranes may not be enough to change the conformation of the spectrum of the base polymer.

Previous studies indicate that large pores, between 250 μm and 500 μm, are more beneficial to cell growth and new matrix production [37]. Oh, et al. (2010) revealed that pores between 300–320 µm and 370–400 µm are more conducive to gene expression of collagen type II and SOX-9, indicators of chondrogenesis, whereas smaller pores favour the production of collagen type I and X [38]. Our morphological results (Table 4), evaluated at 5× magnification, showed filaments with a diameter between 270.14 ± 9.3005 µm (CcC1%) and 326.45 ± 11.674 µm (McC2%) and pore size between 329.72 ± 12.525 µm (CcC2%) and 379.57 ± 24.389 μm (MC2%). Thus, the large pores obtained in our scaffold will be ideal for cell proliferation and infiltration and promote collagen II production, as previous studies have demonstrated. The pore sizes differences between samples are related to the production parameters and not directly to the composites, as these are manually adjusted.

Porosity is related to pore and filament sizes obtained in each scaffold. Although the native porosity values are around 70% to 92% [34], depending on the cartilage zone, the values obtained are a ratio between cell adhesion/proliferation capacity and mechanical properties. When compared with the control (PCL), the samples with higher porosity are McC3%, MC2%, CcC1%, and CcC3% (Table 4). These samples also correspond to the samples with the largest pore size. The remaining samples showed porosities similar to PCL. When comparing the porosity within the same cellulose at different percentages, the scaffold with MC is the only one revealing some variations. The incorporation of 2% of MC leads to an increase in porosity (65.56 ± 1.663%). However, the increase in the percentage leads to a decrease in porosity (59.07 ± 0.8932%). Some studies report increased porosity when MC is increased or no significant change, depending on the method of fabrication of the structure [29,39]. The MC1% and MC3% scaffolds presented similar porosity values in this study. In addition, in these scaffolds, similar pore sizes and filament diameters were obtained, leading to similar porosities.

Despite being lower than the reference values, a previous study of our group reveals that this low porosity does not interfere with cell viability and proliferation of fibroblasts [30]. Additionally, in MicroCT analysis is observable that no agglomerate of cellulose is formed. This result suggests that, although it is not soluble, all celluloses were well dispersed. Finally, regarding interconnectivity, almost all scaffolds presented approximately 100%. This value indicates that cells, nutrients, oxygen, residues, and other biomolecules can migrate freely throughout the whole scaffold [40].

The compression test results (Figure 3) show standard stress–strain thermoplastic curves [41]. The behaviour of the base thermoplastic, PCL, is not affected by the presence of cellulose. However, the mechanical properties of scaffolds are affected by the incorporation of the materials [42]. Compared with the PCL, all scaffolds, except for McC2% and MC1%, possess lower resistance. In general, it is concluded that incorporating cellulose decreases scaffolds’ mechanical properties. Alemán-Dominguez (2018) achieved similar results when incorporating carboxymethyl cellulose into PCL scaffolds [14]. When evaluating the incorporation of cellulose, in McC, the incorporation of 2% did not result in a significant change in its mechanical properties. However, when this percentage is increased to 3%, a decrease in the scaffold properties is revealed. Alemán-Dominguez (2019) found the same effect when increasing the concentration of McC from 2% to 5% [4]. Our study shows that these properties decrease at a lower concentration. Regarding MC, the compression modulus at 2 and 3% were the lower of all samples (Figure 3). The incorporation of 2% immediately results in a decrease from 60.55 ± 3.185 MPa to 31.96 ± 1.565 MPa, almost half of the original properties. When incorporating 3% MC, its resistance is not affected compared to 2%. These values are closer to the desired for application in AC, since the compression modulus for human cartilage varies between 5.5 and 11.8 MPa [43]. CcC scaffolds were the only structure in which their resistance was not affected when CcC was incorporated. These results demonstrate that scaffolds with very similar porosities present similar mechanical properties since McC2% and MC1% present porosity values, 58.47 ± 0.7003% and 57.76 ± 0.4972%, respectively, very close to PCL (57.40 ± 2.616%) (Table 4).

The AC is also subject to tensile forces [44]. For this reason, filaments were subjected to tensile tests (Figure 4). When compared with the PCL scaffold (12.74 ± 1.416 MPa), the MC1%, CcC1%, CcC2%, and CcC3% scaffolds demonstrated the same tensile resistance (15.24 ± 1.017 MPa, 16.05 ± 1.171 MPa, 15.74 ± 1.289 Mpa, and 14.67 ± 0.6385 Mpa, respectively). However, when incorporated McC in all concentrations, or MC at 2% and 3%, the tensile resistance is improved. The highest tensile strength corresponds to the McC2% scaffold (22.84 ± 1.424 MPa). When comparing the incorporation of cellulose, McC demonstrated that the incorporation of 2% improved the tensile force, but at 3%, this resistance decreased (16.94 ± 1.855 MPa). In MC, the incorporation of cellulose is only noticed when incorporated at 3%, with a slight increase in its tensile resistance (18.81 ± 0.5910 MPa). Once again, the increase in CcC does not result in changes in its resistance to this force.

Upon implantation in human/animal tissue, the scaffold is not just susceptible to hydrolytic degradation but also enzymatic. This way, scaffolds were submitted to a degradation test for 42 days (Figure 5). Regarding the base polymer (PCL), it was found that no hydrolytic (PCLh) degradation occurs (Figure 5a). The statistical differences detected in the gain of mass in the first 7 days are related to water absorption by the material. PCL is a hydrophobic polyester [45]. However, processing with DMF leads to an increase in its wettability, making it hydrophilic (ϴ = 88.41°) [46]. Enzymatically the behaviour is similar in the first days, but the scaffold decreases its weight until day 28, stabilising after that. This result shows that, when in contact with lysozyme, PCL starts its degradation at day 7, with a reduction of 0.8% in weight. The scaffold should be degraded and absorbed upon new tissue formation and growth. Thus, the scaffold degradation rate must be inversely proportional to the rate of new tissue formation [47]. We believe that this degradation rate in lysozyme is too fast for cartilage regeneration and, for this reason, may not be a viable solution by itself to be applied in AC regeneration.

When the enzymatic degradation of the scaffolds with cellulose was observed, they presented the same response as the PCL scaffold (Figure 5). Thus, it is concluded that the degradation of the scaffold occurs by the degradation of PCL and not cellulose. At the end of the 42-day assay, McC1%, McC2%, and McC3% had lost about 0.6% of their weight (Figure 5b). CcC1%, CcC2%, and CcC3% lost 0.4%, 0.9%, and 0.7%, respectively (Figure 5d), while MC1%, MC2%, and MC3% lost about 0.8% (Figure 5c), similar to the PCL.

Hydrolytically, the behaviour of McC, MC, and CcC scaffolds is similar to PCL. Thus, once again, results suggest that the changes observed during the test are due to changes in the PCL structure and not in different celluloses, proving that it is not affected by the presence of water. The mass gain in the scaffolds on the first day of the test is due to the hydrolytic property of cellulose [48], enhancing the ability to absorb water. PCL had a gain of 0.6% (Figure 5a). On the other hand, McC2% and MC2% had a similar gain in mass, between 0.5% and 0.8%. The scaffolds with the highest mass gain were CcC1% with 2%, McC1%, McC3%, and CcC3% with 1.2%, and CcC2% with 1.1%. These scaffolds have a greater ability to incorporate water in your matrix and benefit the nutrient change. Scaffolds made with 1 and 3% of MC had a lower water absorption capacity (0.1% and 0.2%, respectively).

On the seventh day of testing, the mass values reached results very similar to those of day 0, as they have reached hydrostatic equilibrium. The scaffold with the slightest mass variation was the McC1%, with a loss of 0.8% of its weight compared to day 1. On day 28 of testing, the mass gain results were not statistically significant for PCL, McC, and CcC. However, when MC was incorporated into the scaffolds, their weight tended to increase, being more noticeable at 2% of MC (about 0.7%). Natural MC presents itself as a gel when in contact with water. Thus, its gelation occurs in the manufacture of the membrane continuing in this state throughout the process. When placed in an aqueous medium (PBS), it tends to absorb part of the medium and, therefore, gain mass [49]. After 42 days of hydrolytic testing, samples that continued to lose weight were MC and CcC. Being a gel, MC is probably more susceptible to starting its degradation. At the time of its extraction, CcC undergoes very aggressive processes [35]. Its structure may no longer be completely intact, and for this reason, it may start its degradation earlier than the other tested celluloses.

A cytocompatibility test was performed using hDPSCs to validate the cellular response of the produced scaffolds (Figure 6). The cytocompatibility was determined at different timepoints using the PrestoBlue^TM^ method. The analysis of the results was carried out following an adaptation of ISO 10993-5:2009 “Biological evaluation of medical devices”—Part 5—“Test for in vitro cytotoxicity”, which has already shown in previous works to guarantee an evaluative performance similar to the MTT assay standardise in the guidelines [32,33]. Unlike the MTT method, Presto Blue is performed on live cells, allowing them to be used over different timepoints, allowing for the use of fewer cells and better assessing cumulative toxicity [50]. hDPSCs were selected as the cell population to be tested due to the prior identification of their ability to induce cartilage tissue regeneration [51]. Following the guidelines of ISO 10993-5:2009, a control group was established using only seeded cells without contact with a scaffold. This group was considered a negative control, representing the cell culture of normal performance without the presence of any cytotoxic or cytoinhibitory effect. Direct contact cytocompatibility was considered, with cells being directly seeded onto the biomaterials and keeping in contact with them in the well. Absorbance values were obtained at each considered timepoint, and the data obtained were analysed according to the manufacturing instructions and presented as corrected absorbances. Subsequently, the same data were normalised considering the absence of toxicity in the control group (0% cell inhibition) and presented as % Viability Inhibition.

In general, the scaffolds proved not to be cytotoxic (Figure 8). However, at some timepoints, some scaffolds are considered cytotoxic. In some scaffolds, this cytotoxicity is reversible, for example, at 24 h in the PCL (borderline values) and McC1% (cytotoxicity indicators). Afterwards, they indicate the absence of cytotoxicity and facilitation of cell proliferation. The McC2% scaffold can be considered the most cytotoxic among those studied since, throughout the entire study period, it always induces inhibition of cell viability with values around 30%. Despite these occasional cases, the percentage of inhibition of cell viability is never particularly high, and it is, even in cases with less favourable results, around the borderline reference value to consider a material as cytotoxic. Most formulations of PCL–cellulose show no cytotoxicity and even values indicative of cell adhesion and proliferation induction. The CcC1% scaffold seems to present the best performance throughout the study, at 24 h showing a clear beneficial effect on cell adhesion and always maintaining negative values of percentage of inhibition of cell viability throughout the study period. Despite the indicative values of some toxicity in the first timepoint, the PCL presents good values in the subsequent phases. At 168 h, practically all formulations show no toxicity, except for McC2% with borderline values (Figure 8).

Accordingly, the absorbance values continuously increase over time for all groups, revealing an increase in cell proliferation while maintaining cell viability (Figure 6). Unexpectedly, at 24 h, the control group is not the one with the highest absorbance values, and others surpass this group over the study period. This result demonstrates that several scaffolds promote cell adhesion and proliferation superior to the ones observed when seeding cells directly into the wells of the plate. Previous studies proved that the presence of cellulose in scaffolds promotes cell adhesion and proliferation, corroborating these results [30].

Cell group was used as a control to determine the percentage of inhibition of cell viability in the remaining groups. So, biomaterials are considered cytotoxic, when absorbance values are noticeably different from the control. However, few statistical differences are observed between groups, and the values indicative of toxicity are always borderline. At 24 h, the only absorbance values different from PCL were McC1%, McC2%, and CcC1% (Figure 6). All other scaffolds behave similarly to PCL in terms of cell adhesion. However, at 168 h, the only samples with absorbance values similar to PCL were McC3% and CcC1%. For MC, the incorporation of cellulose does not translate into significant changes in the absorbance value at 24 h nor over the cell assay time. However, for McC, the absorbance values of McC3% increase from 24 h to 168 h. As for CcC, the absorbance values of CcC1% are always higher than the other percentages. Scaffolds with the best absorbances are the CcC1% and McC3% along the different timepoints, and the PCL at the latter. These values corroborate those observed in evaluating the percentage of inhibition of viability.

Once the compatibility of the manufactured scaffolds is confirmed and considering the results obtained in the different assays, the best PCL–cellulose combinations will be submitted to chondrogenic differentiation assays to determine the ability of hDPSCs to differentiate in the presence of these biomaterials. The ability of cells to adhere to scaffolds will be attested by scanning electron microscopy assays, and changes in gene expression associated with genes influencing chondrogenic differentiation will be determined by RT-PCR assays. After these phases and confirming favourable results, the manufactured scaffolds could be applied in vivo to demonstrate that they are suitable for promoting cartilaginous regeneration.

## 5. Conclusions and Future Work

PCL and PCL–cellulose scaffolds were developed to evaluate their potential for the regeneration of damaged cartilage. Morphologically, manufactured scaffolds are very similar to each other. However, the incorporation of cellulose leads to an increase in the porosity of the scaffolds. Mechanically, this increase in porosity makes its resistance decrease. However, obtained values are within the expected and required for potential application in cartilage regeneration. Regarding the elasticity of the scaffold filaments, these were revealed to be more elastic upon cellulose incorporation, being the McC2% scaffold the most elastic. When submitted to enzymatic and hydrolytic degradation, it was verified that the PCL scaffolds suffered degradation after 7 days. This degradation is also observed in scaffolds with different incorporated celluloses. When their adhesion capacity and cell proliferation were evaluated, the scaffolds with the best results were PCL, McC3%, and CcC1%. In general, regarding the properties assessed in this study, scaffolds manufactured with PCL-MC 2% and 3% prove to be more advantageous to proceed to further studies for cartilage repair application. These scaffolds present compressive performance more similar to native cartilage, and all biological studies assure good cell adhesion and proliferation. In enzymatic tests, it behaves as a hydrogel, soft material, corroborating the “bumper” behaviour of the cartilage tissue. Regarding the ideal percentage, further studies are still required.

The outcomes observed in this work are thus a starting point for future tests, where the main aim will be to apply the characterized biomaterials in the promotion of cartilage regeneration and repair. The results obtained here allow us to understand the degree of toxicity induced by direct contact between the hDPSCs and the scaffolds, but more specific tests will be necessary to effectively determine the permissibility of these biomaterials in terms of cell adhesion on their surface and the proliferation into the pores. This adhesion and proliferation must thus be confirmed by scanning electron microscopy after a new culture by direct contact. Likewise, the ability of the scaffolds to induce chondrogenic differentiation when in contact with the cells still needs to be determined, and this should be tested both qualitatively and quantitatively by measuring the production of proteoglycans and by identifying variations in gene expressions associated with this differentiation by RT-PCR. This whole set of new tests is planned to determine the best scaffold to be applied in in vivo tests, thus allowing to confirm its effectiveness in promoting successful cartilaginous regeneration.

## Figures and Tables

**Table 1:** Nomenclature used for the different PCL and cellulose (microcrystalline, methyl-, and corncob) samples, with respective percentage and type of cellulose.

Base-Polymer	Type/Origin of Cellulose	Percentage of Cellulose	Nomenclature
PCL	-	-	PCL
	Microcrystalline	1%	McC1%
		2%	McC2%
		3%	McC3%
	Methyl	1%	MC1%
		2%	MC2%
		3%	MC3%
	Corncob	1%	CcC1%
		2%	CcC2%
		3%	CcC3%

**Table 2:** Parameters of the equipment used in the extrusion of each PCL–cellulose mixture.

Sample	Crosshead Speed (mm/s)	Deposition Spindle Speed (rpm)	Nozzle Temperature (°C)
PCL	8	15.33	76
McC1%	8	12.33	84
McC2%	8	12.33	84
McC3%	7	13	85
MC1%	7	13.67	86
MC2%	7	14	85
MC3%	7	14.17	86
CcC1%	7	12.83	86
CcC2%	7	13.83	86
CcC3%	7	13.33	85

**Table 3:** Filament diameter (mean ± SD) manufactured for mechanical tensile test.

Sample	Filament Diameter (µm)
PCL	0.499 ± 0.016
McC1%	0.533 ± 0.005
McC2%	0.569 ± 0.019
McC3%	0.529 ± 0.007
MC1%	0.502 ± 0.008
MC2%	0.520 ± 0.010
MC3%	0.549 ± 0.041
CcC1%	0.506 ± 0.006
CcC2%	0.549 ± 0.013
CcC3%	0.559 ± 0.012

**Table 4:** Filament and pore dimensions, porosity, and interconnectivity of the scaffolds (mean ± SD) assessed through micro-computed tomography (MicroCT).

Sample	Filament (µm)	Pore (µm)	Porosity (%)	Interconnectivity (%)
PCL	324.31 ± 11.540	344.25 ± 16.391	57.40 ± 2.616	99.993 ± 0.00404
McC1%	309.40 ± 10.960	350.47 ± 21.683	59.10 ± 0.4051	100.000 ± 0.00058
McC2%	326.45 ± 11.674	344.27 ± 11.077	58.47 ± 0.7003	99.995 ± 0.00529
McC3%	303.92 ± 13.713	357.36 ± 16.376	62.36 ± 1.448 ^a^	99.097 ± 1.5630
MC1%	305.89 ± 14.711	356.10 ± 21.137	57.76 ± 0.4972 ^b^	99.994 ± 0.00872
MC2%	281.66 ± 14.610	379.57 ± 24.389	65.56 ± 1.663 ^a,b,c^	99.999 ± 0.00153
MC3%	306.12 ± 8.6990	354.75 ± 11.359	59.07 ± 0.8932 ^c^	99.999 ± 0.00058
CcC1%	270.14 ± 9.3005	369.53 ± 16.292	63.56 ± 1.501 ^a^	99.997 ± 0.00379
CcC2%	292.11 ± 9.3061	329.72 ± 12.525	61.36 ± 2.510	99.998 ± 0.00321
CcC3%	286.46 ± 17.991	356.21 ± 19.100	62.65 ± 0.8638 ^a^	99.998 ± 0.00100

a—statistical difference with control (PCL). b, c—statistical difference between same samples, different percentage.

## Data Availability

Not applicable.

## Supplementary Materials

The following supporting information can be downloaded at: https://www.mdpi.com/article/10.3390/polym15030781/s1, Table S1: Cytocompatibility assessed by PrestoBlue^TM^ viability assay with hDPSCs. Corrected absorbance results are presented in Mean ± SEM: Table S2: Percentage of inhibition of cell viability of each scaffold in contact with the hDPSCs. After normalisation of the Control group (100%), results are presented as Mean ± SEM for the percentage of viability inhibition, comparing to the control group; Figure S1: Percentage of inhibition of cell viability of each scaffold in contact with the hDPSCs at 24 h. The dashed line represents the 30% inhibition limit, above which the effect is considered cytotoxic according to the ISO 10993-5:2009 guidelines; Figure S2: Percentage of inhibition of cell viability of each scaffold in contact with the hDPSCs at 72 h. The dashed line represents the 30% inhibition limit, above which the effect is considered cytotoxic according to the ISO 10993-5:2009 guidelines; Figure S3: Percentage of inhibition of cell viability of each scaffold in contact with the hDPSCs at 120 h. The dashed line represents the 30% inhibition limit, above which the effect is considered cytotoxic according to the ISO 10993-5:2009 guidelines; Figure S4: Percentage of inhibition of cell viability of each scaffold in contact with the hDPSCs at 168 h. The dashed line represents the 30% inhibition limit, above which the effect is considered cytotoxic according to the ISO 10993-5:2009 guidelines.
