# Supplementary material for: Cellulose-Based Scaffolds: A Comparative Study for Potential Application in Articular Cartilage
Source: Polymers (Basel). 2023 Feb 3;15(3):781. doi: 10.3390/polym15030781 (PMC9919712; doi:10.3390/polym15030781)
Supplement: Supplementary file 1 [file polymers-15-00781-s001.zip › polymers-2045773-supplementary.pdf]

Supplementary Material

Table S1- Cytocompatibility assessed by Presto Blue™ viability assay with hDPSCs. Corrected absorbance results are presented in Mean ± SEM.

Timepoint / Scaffold	24h	72h	120h	168h
PCL	0.025 ± 0.005	0.0378 ± 0.003	0.040 ± 0.004	0.079 ± 0.044
McC1%	0.012 ± 0.001	0.028 ± 0.003	0.027 ± 0.002	0.054 ± 0.005
McC2%	0.010 ± 0.001	0.021 ± 0.000	0.017 ± 0.002	0.040 ± 0.005
McC3%	0.023 ± 0.001	0.047 ± 0.002	0.052 ± 0.002	0.067 ± 0.002
MC1%	0.016 ± 0,002	0.028 ± 0.002	0.030 ± 0.003	0.042 ± 0.010
MC2%	0.020 ± 0004	0.023 ± 0.002	0.033 ± 0.004	0.050 ± 0.005
MC3%	0.025 ± 0.001	0.029 ± 0.001	0.049 ± 0.005	0.056 ± 0.004
CcC1%	0.042 ± 0.005	0.0480 ± 0.007	0.038 ± 0.008	0.066 ± 0.004
CcC2%	0.022 ± 0.001	0.026 ± 0.001	0.031 ± 0.002	0.044 ± 0.004
CcC3%	0.019 ± 0.001	0.031 ± 0.002	0.047 ± 0.003	0.051 ± 0.004
Cells	0.021 ± 0.001	0.031 ± 0.001	0.036 ± 0.001	0.055 ± 0.003

Table S2- Percentage of inhibition of cell viability of each scaffold in contact with the hDPSCs. After normalization of the Control group (100%), results are presented as Mean ± SEM for the percentage of viability inhibition, comparing to the control group.

Timepoint / Scaffold	24h	72h	120h	168h
PCL	28.84 ± 11.70	-7.61 ± 6.62	-10.36 ± 10.75	-94.50 ± 18.14
McC1%	43.53 ± 4.23	13.39 ± 8.70	13.22 ± 7.30	4.61 ± 0.22
McC2%	46.75 ± 2.32	31.81 ± 1.05	21.93 ± 1.61	31.19 ± 0.25
McC3%	-12.55 ± 5.25	-18.11 ± 0.00	-30.11 ± 2.31	-71.84 ± 11.18
MC1%	24.16 ± 7.37	12.34 ± 5.21	15.43 ± 9.58	-70.39 ± 17.35
MC2%	5.20 ± 20.91	7.09 ± 4.23	8.46 ± 10.83	-41.92 ± 17.08
MC3%	-20.61 ± 3.48	-10.24 ± 0.00	-35.46 ± 14.51	-63.77 ± 16.09
CcC1%	-104.10 ± 24.49	-51.44 ± 23.27	-71.47 ± 15.18	-69.96 ± 8.29
CcC2%	-7.71 ± 4.44	-24.41 ± 13.84	-28.03 ± 8.98	-18.29 ± 22.68
CcC3%	6.82 ± 6.61	2.62 ± 5.79	-30.58 ± 8.22	-19.86 ± 4.20

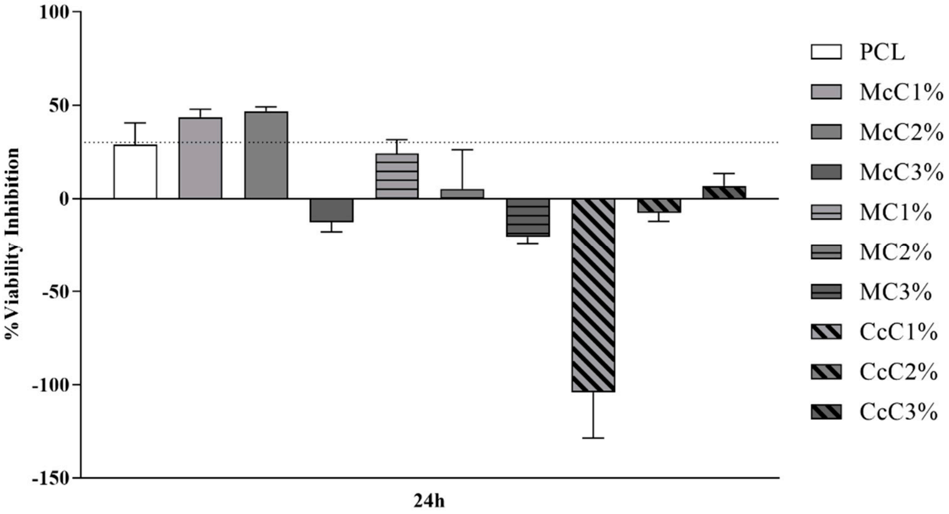

Figure S1- Percentage of inhibition of cell viability of each scaffold in contact with the hDPSCs at 24h. The dashed line represents the 30% inhibition limit, above which the effect is considered cytotoxic according to the ISO 10993-5:2009 guidelines.

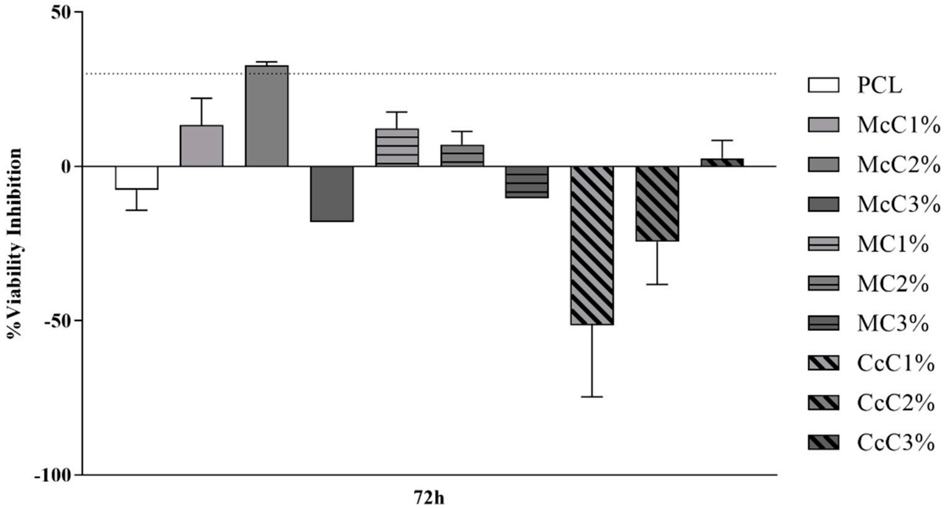

Figure S2- Percentage of inhibition of cell viability of each scaffold in contact with the hDPSCs at 72h. The dashed line represents the 30% inhibition limit, above which the effect is considered cytotoxic according to the ISO 10993-5:2009 guidelines.

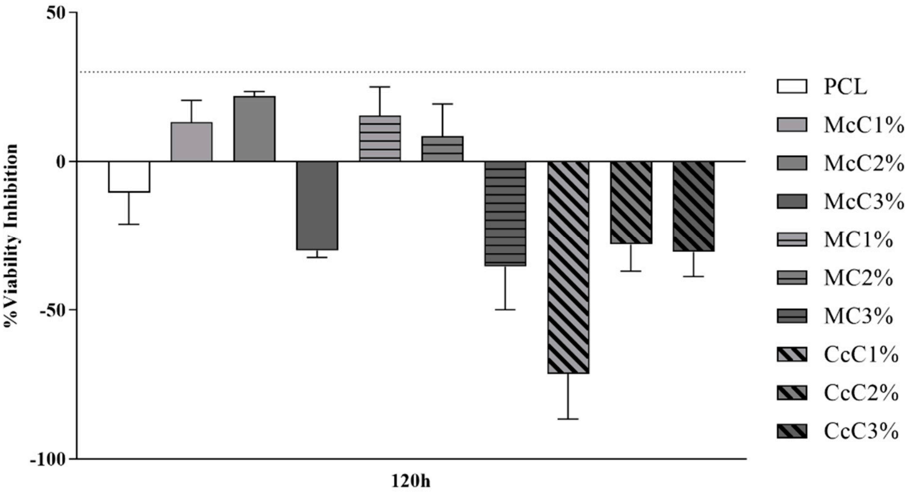

Figure S3- Percentage of inhibition of cell viability of each scaffold in contact with the hDPSCs at 120h. The dashed line represents the 30% inhibition limit, above which the effect is considered cytotoxic according to the ISO 10993-5:2009 guidelines.

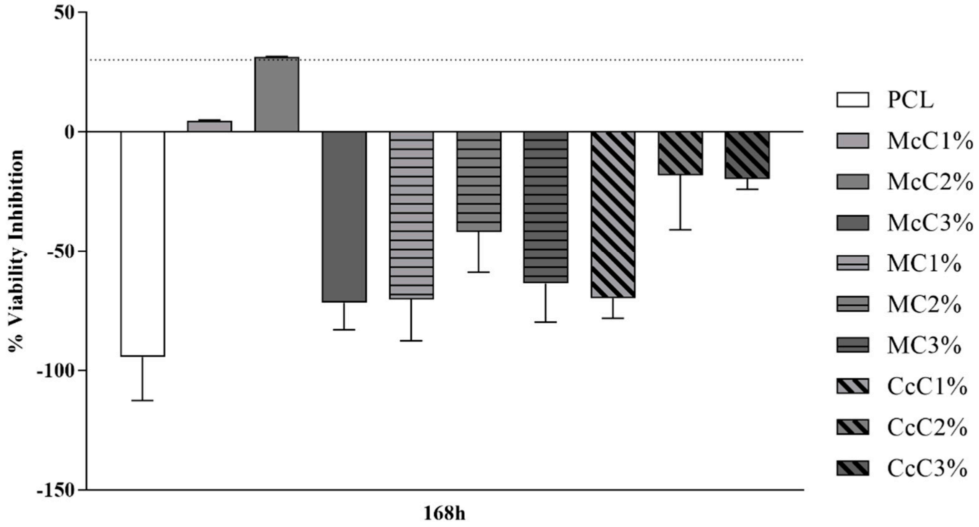

Figure S4- Percentage of inhibition of cell viability of each scaffold in contact with the hDPSCs at 168h. The dashed line represents the 30% inhibition limit, above which the effect is considered cytotoxic according to the ISO 10993-5:2009 guidelines.
